# Examining the patterns and dynamics of species abundance distributions in succession of forest communities by model selection

**DOI:** 10.1371/journal.pone.0196898

**Published:** 2018-05-10

**Authors:** Zuo-Yun Yin, Lu Zeng, Shao-Ming Luo, Ping Chen, Xiao He, Wei Guo, Bailian Li

**Affiliations:** 1 College of Horticulture and Landscape Architecture, Zhongkai University of Agriculture and Engineering, Guangzhou, Guangdong, China; 2 Guangdong Provincial Key Laboratory of Forest Breeding, Protection and Utilization, Guangzhou, Guangdong, China; 3 Guangdong Forest Research Institute (Guangdong Academy of Forestry), Guangzhou, Guangdong, China; 4 South China Botanical Garden of the Chinese Academy of Sciences, Guangzhou, Guangdong, China; 5 Ecological Complexity and Modeling Laboratory, Department of Botany and Plant Sciences, University of California, Riverside, California, United States of America; 6 Guangdong Ecological Engineering Vocational College, Guangzhou, Guangdong, China; 7 Faculty of Mechanical and Electrical Engineering, Zhongkai University of Agriculture and Engineering, Guangzhou, Guangdong, China; 8 College of Economics, Guangdong University of Finance and Economics (formerly Guangdong University of Business Studies), Guangzhou, Guangdong, China; University of South Carolina, UNITED STATES

## Abstract

There are a few common species and many rare species in a biological community or a multi-species collection in given space and time. This hollow distribution curve is called species abundance distribution (SAD). Few studies have examined the patterns and dynamics of SADs during the succession of forest communities by model selection. This study explored whether the communities in different successional stages followed different SAD models and whether there existed a best SAD model to reveal their intrinsic quantitative features of structure and dynamics in succession. The abundance (the number of individuals) of each vascular plant was surveyed by quadrat sampling method from the tree, shrub and herb layers in two typical communities (i.e., the evergreen needle- and broad-leaved mixed forest and the monsoon evergreen broad-leaved forest) in southern subtropical Dinghushan Biosphere Reserve, South China. The sites of two forest communities in different successional stages are both 1 ha in area. We collected seven widely representative SAD models with obviously different function forms and transformed them into the same octave (log_2_) scale. These models are simultaneously confronted with eight datasets from four layers of two communities, and their goodness-of-fits to the data were evaluated by the chi-squared test, the adjusted coefficient of determination and the information criteria. The results indicated that: (1) the logCauchy model followed all the datasets and was the best among seven models; (2) the fitness of each model to the data was not directly related to the successional stage of forest community; (3) according to the SAD curves predicted by the best model (i.e., the logCauchy), the proportion of rare species decreased but that of common ones increased in the upper layers with succession, while the reverse was true in the lower layers; and (4) the difference of the SADs increased between the upper and the lower layers with succession. We concluded that the logCauchy model had the widest applicability in describing the SADs, and could best mirror the SAD patterns and dynamics of communities and their different layers in the succession of forests. The logCauchy-modeled SADs can quantitatively guide the construction of ecological forests and the restoration of degraded vegetation.

## Introduction

There are a few common species and many rare species in a biological community (or a multi-species collection) in given space and time. This hollow (i.e., inverse J-shaped, hyperbolic) distribution curve of the species number on the individual number (abundance) in the community is called “species abundance distribution” (SAD) [1–9]. The patterns in the distribution and abundance of species within a biome are central concerns in ecology, as they provide important information about the total species richness, the species-area relation, succession, the likelihood of species extinction under habitat loss, the reserve design, and the processes that allow species to coexist and partition resources [10–13]. This pattern is so fundamental that Sugihara (1980) referred to it as “minimal community structure” [14].

Ecologists have proposed many models describing the SADs in various communities, including the empirical models such as logseries (LSer) [1] and lognormal distribution (LN) [2], and the theoretical models such as geometric series (GS; or niche pre-emption) [15], broken stick (BS) and overlapping niche (ON) [16]. The empirical models have been applied to describe the SAD, while the theoretical ones to describe the curve of the individual number (ranked from most to least, or vice versa) on the species number, i.e., the rank abundance curve (RAC) [11–12,17–20].

Many have conducted a large number of case studies to model the SADs [2,11,14,16, 21–27]. However, little effort has been made to find out the best among these SAD models through the “model selection” method instead of commonly-used zero hypothesis method [28]. But Yin (2005) proposed a “sequential distribution set” by integrating logCauchy (LC) and log-sech (LS) distributions with the widely used lognormal (LN), and modeled the species abundance data from six forest communities (five in lower subtropics and one in tropics) [22,29]. He found that the LC was the best although the three models statistically adequately fitted all the datasets. Recently, Wei et al. (2014) explored the SAD models that are appropriate for six subtropical forests [30]. They used three models, namely the lognormal, logCauchy and logseries, to fit the datasets from six communities, and similarly concluded that the logCauchy model had the best fitness based on the coefficient of determination.

On the other hand, less effort has been made to examine the relationships between the SADs and the succession of communities by using the best model. But Whittaker (1972) evaluated the changes of SAD during the succession from waste farmland to oak forest and concluded that the SAD followed the GS in early stage and the LN in late stage [31]. Yin et al. (2009) selected seven widely representative SAD models to comparatively explore the SAD patterns and dynamics during the natural restoration of degraded hilly grassland in southern China [32]. Their results showed that: (1) the logCauchy distribution best followed the data; (2) the fit of each model to observation was not directly related to successional stage; and (3) the commonness and rarity of species changed with succession. Are these true or false for the forest land, especially in tropics and subtropics?

Here we try to answer the following questions: (1) whether forest communities in different successional stages in lower subtropics follow different SAD models; (2) whether there is a best SAD model in a widely representative model set; and (3) whether the best model can reveal the intrinsic quantitative features of community structure and dynamics in succession.

## Materials and methods

### Community survey

We selected two 1-ha (100 m×100 m) permanent forest sites (plots) at Dinghushan Forest Ecosystem Research Station (geographic coordinates: 112°30'39''-112°33'41'' E, 23°09'21''-23°11'30'' N; see websites: <http://dhf.cern.ac.cn/content?id=16383> and <http://dhf.cern.ac.cn/contentpic?id=36780>) in Dinghushan Biosphere Reserve, Zhaoqing, Guangdong of China. The two sites established in 1999 are about 2 km apart, and represent two typical forest communities in different successional stages [33]: 70~80-year-old evergreen needle- and broad-leaved mixed forest at Feitianyan, and >400-year-old monsoon evergreen broad-leaved forest at Sanbaofeng. The former is dominated by the tree species *Schima superba*, *Castanopsis chinensis* and *Pinus massoniana*, while the latter by *Cryptocarya concinna*, *C*. *chinensis* and *Aporosa yunnanensis*.

The abundance (i.e., the number of individuals) of each vascular plant for each plot was investigated by quadrat sampling method. The trees (diameter at breast height, DBH ≥1 cm) were surveyed in the tree layer at 25 adjacent quadrats of 20 m×20 m. Each such quadrat was divided into 16 sub-quadrats (5 m×5 m), one of which was then randomly chosen to record the shrubs and the seedlings of trees (DBH <1 cm, height ≥50 cm) in the shrub layer. Meanwhile, within each of the above-chosen 25 sub-quadrats, a 1 m×1 m quadrat was then randomly selected to count the herbs and the woody seedlings (height <50 cm) in the herb layer.

### Model set

Using the method of model selection [28], we assembled the GS, BS, ON and LSer models into the sequential distribution set of the LC+LS+LN to describe the SADs [22,32]. The GS, BS and ON are theoretical models, while the LSer, LC, LS and LN are empirical ones. As mentioned below, the seven models are widely representative with obviously different function types on log scale. They were simultaneously confronted with eight datasets from four layers of two forest communities in different successional stages in South China.

For the model of geometric series (GS, or niche preemption) [31], the abundance proportion of *j*th species is given by

$$p_{j}=k{\left(1-k\right)}^{j-1}$$ (1)

where 0 < *k* < 1; *j* = 1, 2, …, *S* (the total number of species in a community). This is the RAC model of GS, which is equivalent to the SAD model of truncated hyperbola [13], so the number of species that represents *r* individuals is written as

$$S(r)=\frac{C}{r}$$ (2)

where *r*∈[*r*_min_, *r*_max_]; *r*_min_ and *r*_max_ are the smallest and largest abundance, respectively; and *C* is a constant. On log_2_ scale, Eq 2 can be transformed into

$$S(R)=S_{m}$$ (3)

where *R* is the “octave” called by Preston (1948) [2], equivalent to log_2_ scale of *r*, i.e., *R* = log_2_*r*; *S*(*R*) is the number of species in *R*th octave; *S*_m_ is a constant.

As to the broken stick (BS) model [16], when the species are arranged from the most common to the rarest, the proportional number of individuals of *j*th most common species is

$$p_{j}=\frac{1}{S}\sum_{i=j}^{S}\frac{1}{i}$$ (4)

where *j* = 1, 2, …, *S*. The above RAC-typed BS model and the SAD-typed exponential distribution model are interdeducible [34]. The latter is as follows,

$$S(r)=Ce^{-\alpha r}$$ (5)

where *r*∈[0, ∞); *C* is a constant. Correspondingly, SAD model on log_2_ scale is given by

$$S(R)=S_{m}\exp(-2^{R}\alpha +R\ln2)$$ (6)

where *R*∈(–∞, ∞); *S*_m_ and α are constants [32].

We can estimate the theoretical total number of species in the whole community, S*, by extrapolating the curve below the minimal abundance class and measuring the area below the complete (non-truncated) curve [2,21,29]. For Eq 6, we have [32]

$$S^{*}\approx \frac{S_{m}}{\alpha \ln2}$$ (7)

In the overlapping niche (ON) model [16], the relative abundance per species follows a linear distribution [35]:

f(r)=2−2r (8)

where *r*∈[0, 1]. The corresponding SAD model on log_2_ scale was derived [32] as

$$S(R)=S_{m}\left(1-\frac{2^{R}}{N_{\left(R\right)}}\right)2^{R}$$ (9)

where *R*∈(–∞, *R*_max_], *R*_max_ is the observed largest octave; *S*_m_ is a constant; *N*_(*R*)_ is the total number of individuals calculated after octave transformation, i.e., *N*_(*R*)_ = Σ2^*R*^*S*_obs_(*R*), within which *S*_obs_(*R*) is the observed number of species in *R*th octave. Similarly, we can get the estimation of S* [32],

$$S^{*}\approx \frac{S_{m}}{2\ln2}$$ (10)

For the logseries (LSer) model derived by Fisher et al. (1943) [1], the expected number of species having *r* individuals is given by

$$S_{r}=C\frac{\alpha^{r}}{r}$$ (11)

where *r* = 1, 2, 3, …; α∈(0, 1); *C* is a constant. Plotkin & Muller-Landau (2002) proposed a corresponding distribution density function [13],

$$f(r)=\frac{\alpha^{r}}{r\Gamma (0,-\ln\alpha )}$$ (12)

where *r*∈[1, ∞). We can transform the function into the SAD model on log_2_ scale [32],

$$S(R)=S_{m}\alpha^{2^{R}}$$ (13)

where *R*∈[0, ∞). The LSer cannot estimate the S* [21].

The general equation of the lognormal (LN) distribution model is

$$S(R)=S_{m}\exp[-\alpha^{2}{\left(R-R_{m}\right)}^{2}]$$ (14)

where *R*∈(–∞, ∞); *R*_m_ is the modal (peak) octave; *S*_m_ is the number of species in the *R*_m_th octave (or the height of the curve); *α* is a constant [2]. Preston (1948) first proposed the estimation of S*,

$$S^{*}\approx \frac{\sqrt{\pi }S_{m}}{\alpha }$$ (15)

Yin (2005) first applied a logCauchy (LC) distribution to community ecology for modeling the observed SADs in forest communities [22]. The LC model on log_2_ scale is

$$S(R)=\frac{S_{m}}{1+\alpha^{2}{\left(R-R_{m}\right)}^{2}}$$ (16)

where three parameters, *S*_m_, α, and *R*_m_, are similar to the LN’s counterparts. Similarly, the S* can be estimated by [22,29]

$$S^{*}\approx \frac{\pi S_{m}}{\alpha }$$ (17)

Yin (2005) also first proposed a log-sech (LS) distribution to describe the species abundance data [22], given by

$$S(R)=S_{m}\mathrm{sech}[\alpha (R-R_{m})]$$ (18)

where *S*_m_, α, and *R*_m_ are similar to the LN’s and LC’s counterparts. In the same way, we can deduce the S* calculating formula [22,29],

$$S^{*}\approx \frac{\pi S_{m}}{\alpha }$$ (19)

### Data analysis

To ensure the comparability of data analyses, we used the same methods for quadrat-sampling and abundance-grouping between two communities, and the same methods for fitting, testing and analyzing among seven models. The abundances for each dataset were grouped by Preston’s (1948) octave method [2,36]. The seven models were defined as Eqs (3), (6), (9), (13), (14), (16) and (18) for the GS, BS, ON, LSer, LN, LC, and LS models, respectively. Model parameters were estimated by using Levenberg-Marquardt least squared nonlinear regression of *S*(*R*) against *R* by SPSS 9.0 (SPSS Inc.). We measured the significance of model fitting to data by the chi-square test at 0.05 level. Simultaneously, we evaluated the goodness-of-fit among models by the criterion of the coefficients of determination (i.e., *R*_d_^2^ ≥ 0.50) of the regression [29,37–38], as well as the following Akaike Information Criterion (AIC) and Bayesian Information Criterion (BIC) [39–42],

$$AIC=nln\left(SS_{Residual}\right)+2k-nln\left(n\right)$$ (20)

$$BIC=nln\left(SS_{Residual}\right)+kln\left(n\right)-nln\left(n\right)$$ (21)

where *n* is sample size (here the number of observed octaves); *k* is the number of parameters; and SS_Residual_ is the residual sum of squares. In contrast to the *R*_d_^2^, the lower the AIC or BIC, the better the model.

## Results

### Species abundance data

The evergreen needle- and broad-leaved mixed forest and the monsoon evergreen broad-leaved forest typically represented two different successional stages in southern subtropical Dinghushan Biosphere Reserve, South China. The latter had more species than the former in each community layer (Table 1). With the succession of the mixed to the broad-leaved, for the species number, the upper layers (pure tree layer and tree layer) increased and the lower layers (shrub layer and herb layer) increased much more; while for the individual number, the upper changed not significantly but the lower more than doubled.

**Table 1:** Descriptive statistics of species abundance data from four layers of two forest communities in Dinghushan Biosphere Reserve, Guangdong of China.

Layer / Community	Total area (m^2^)	Total species number, *S*	Total individual number, *N*	Min. individual number	Max. individual number
Pure tree, DH	10000	59	2069	1	573
Tree, DH	10000	71	3899	1	773
Shrub, DH	625	39	433	1	83
Herb, DH	25	32	150	1	29
Pure tree, DK	10000	80	2412	1	848
Tree, DK	10000	88	3382	1	995
Shrub, DK	625	94	936	1	238
Herb, DK	25	47	437	1	217

DH: evergreen needle- and broad-leaved mixed forest at Feitianyan; DK: monsoon evergreen broad-leaved forest at Sanbaofeng; Pure tree layer: *H* ≥ 3 m; Tree layer: DBH ≥ 1 cm; Shrub layer: DBH < 1 cm and *H* ≥ 50 cm; Herb layer: H < 50 cm (These are the same elsewhere).

### Models’ fitting and testing

**Fig 1:**
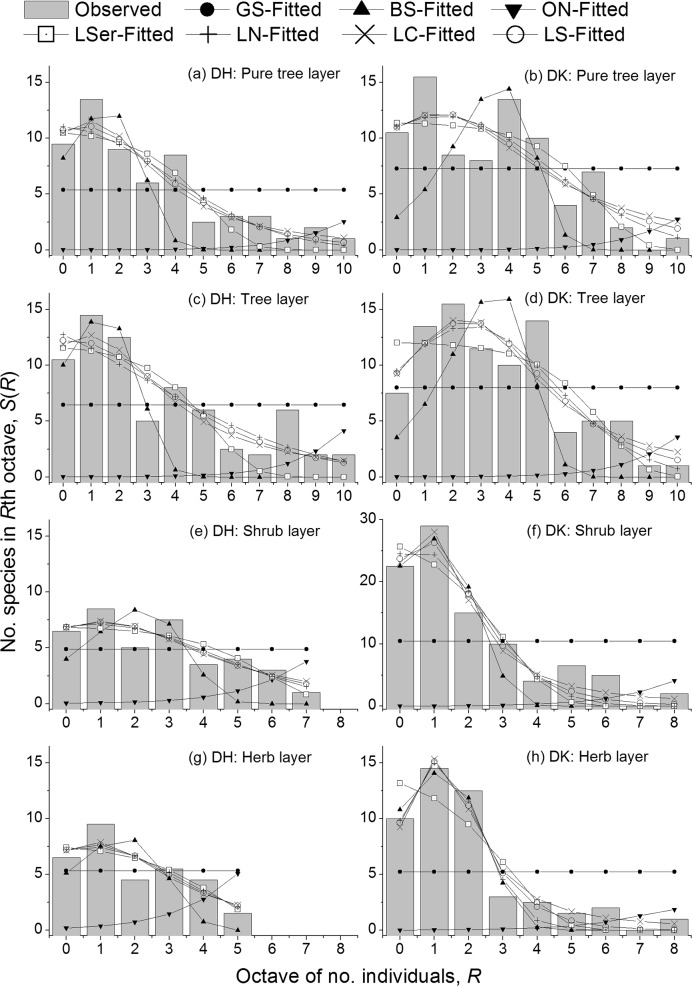
Observed and fitted species-abundance distributions (SADs) in respective four layers within the two communities in Dinghushan Biosphere, Guangdong of China. (Observed: the observed number of species; Fitted: the model-expected number of species. DH: evergreen needle- and broad-leaved mixed forest at Feitianyan; DK: monsoon evergreen broad-leaved forest at Sanbaofeng).

The observed SADs each showed a left-truncated peak shape (bell shape) with the modal octave *R*_m_ = 1, except *R*_m_ = 2 for the tree layer of the broad-leaved forest, in four layers of two forest communities (Fig 1). This suggested that each layer of two communities had many rare species and a few common species. The Chi-square test probability *P*(χ^2^), the determination coefficient *R*_d_^2^, and two information criteria AIC & BIC generally suggested that the LC model followed all the eight datasets, and moreover, it was the best in the set of seven models (Fig 1; Tables 2–4).

**Table 2:** Comparisons of the goodness-of-fit of seven SAD models to species abundance data from four layers of two forest communities in Dinghushan Biosphere Reserve, Guangdong of China.

Layer / Community	GS	BS	ON	LSer	LN	LC	LS
Pure tree, DH	0.000	0.351	–1.748	0.746	0.804*	0.845*	0.829*
Tree, DH	0.000	0.175	–2.194	0.532	0.670*	0.736*	0.700*
Shrub, DH	0.000*	–0.270	–3.863	0.700*	0.677*	0.662*	0.672*
Herb, DH	0.000*	–0.270	–3.869	0.598*	0.481*	0.504*	0.493*
Pure tree, DK	0.000	–0.102	–2.218	0.732*	0.668*	0.600*	0.632*
Tree, DK	0.000	0.053	–2.529	0.677*	0.759*	0.745*	0.759*
Shrub, DK	0.000	0.803	–1.232	0.821	0.850	0.949*	0.905
Herb, DK	0.000	0.930	–0.984	0.802	0.929*	0.940*	0.939

Values in this table are coefficients of determination (*R*_d_^2^)

*: model statistically significantly agrees with data by using the chi-squared goodness-of-fit test at 0.05 level.

**Table 3:** Comparisons of AICs for seven SAD models fitting the species abundance data from four layers of two forest communities in Dinghushan Biosphere Reserve, Guangdong of China.

Layer / Community	GS	BS	ON	LSer	LN	LC	LS
Pure tree, DH	32.5	28.5	43.6	18.2	16.1	13.5	14.6
Tree, DH	33.8	32.5	46.6	26.3	23.1	20.7	22.1
Shrub, DH	15.6	18.3	28.3	6.7	7.9	8.2	8.0
Herb, DH	12.6	14.6	22.1	7.8	9.5	9.3	9.4
Pure tree, DK	36.7	38.6	49.5	23.0	26.1	28.1	27.2
Tree, DK	37.2	37.5	51.1	25.6	23.2	23.8	23.1
Shrub, DK	42.1	28.3	49.4	27.5	26.5	16.8	22.3
Herb, DK	31.7	8.6	37.8	17.9	9.3	7.8	7.9

**Table 4:** Comparisons of BICs for seven SAD models fitting the species abundance data from four layers of two forest communities in Dinghushan Biosphere Reserve, Guangdong of China.

Layer / Community	GS	BS	ON	LSer	LN	LC	LS
Pure tree, DH	32.9	29.3	44.0	19.0	17.3	14.7	15.8
Tree, DH	34.2	33.3	47.0	27.1	24.3	21.9	23.3
Shrub, DH	15.7	18.5	28.3	6.9	8.1	8.5	8.2
Herb, DH	12.3	14.2	21.8	7.3	8.9	8.7	8.8
Pure tree, DK	37.1	39.4	49.9	23.8	27.3	29.3	28.4
Tree, DK	37.6	38.3	51.5	26.4	24.3	24.9	24.3
Shrub, DK	42.3	28.7	49.6	27.9	27.1	17.3	22.9
Herb, DK	31.9	9.0	38.0	18.3	9.9	8.3	8.5

Firstly, we examined the goodness-of-fit of model to data by using chi-square test (Table 2). If the *P*(χ^2^) ≥ 0.05, then distribution model confirms with the observation. The LC model followed all the eight datasets, the LN seven, the LS six, the LSer the half, and the GS only two, while the BS and ON models did not all.

The LC also well followed all according to the *R*_d_^2^ criterion (Table 2). Although the LSer followed all, its *R*_d_^2^ values were lower than the counterparts of LN’s, LC’s or LS’s in most cases. The LN or LS did not follow only one dataset (the herb layer of mixed forest). The order of magnitude in *R*_d_^2^ generally was: LN < LS < LC. The GS model has no variables and thus its *R*_d_^2^ values all were zero. The BS followed only two datasets, while the ON did not follow all due to its negative *R*_d_^2^ values in all cases.

Finally, we compared the seven models by using two information criteria (Tables 3 and 4). For either AIC or BIC, the LSer, LN, LC and LS generally were close to each other, and much lower than GS, BS and ON. In five out of eight cases, the LC was less than the LSer, LN or LS for each of the information criteria, while in other cases, the former was slightly greater than the latter. Similarly to *R*_d_^2^, both AIC and BIC proved that the LC were the best among the seven models.

The fitness of models to the observations was not directly related to the successional stage of community forest. This is because in those scenarios that models were conformed with data, there were the different layers of communities in either early or late successional stages. The GS model by *P*(χ^2^) followed the datasets only from the shrub and herb layers of mixed forest in early successional stage, while the BS by *R*_d_^2^ followed the datasets only from the same two layers of broad-leaved forest in late successional stage. On the other hand, either by *P*(χ^2^) or *R*_d_^2^, the ON could not describe all the communities either in early or late successional stages, while the LC, LS, LN and LSer could describe most by *P*(χ^2^) and all by *R*_d_^2^.

### Predictions and comparisons of S*

In the case that the observed SADs were left-truncated distributions, the BS, ON, LN, LC or LS model could be used to calculate the S* (Table 5). The S* values predicted by the LN, LC and LS were larger than the corresponding *S* values, with an exception of the LN-predicted S* for the herb layer of broad-leaved forest. The BS-predicted S* values were greater than the corresponding *S* values only in three out of eight cases, while the ON-predicted S* values were lower than the corresponding *S* values in all the cases. For the estimations of S*, the LN, LC and LS models were relatively reasonable (i.e., S* > *S*). However, the LC only had the increasing trend for each of four layers from the mixed to the broad-leaved forest, while both the LN and LS showed much decreasing trend for the tree layer, and slightly decreasing trend for the herb layer. These different performances of five models mentioned above were obviously related to their goodness-of-fits to the data. It was proved from the other side that the LC was the best model.

**Table 5:** Comparisons of the observed and five-SAD-model-expected total numbers of species in four layers of two communities in Dinghushan Biosphere Reserve, Guangdong of China.

Layer / Community	*S*	S^*^_BS	S^*^_ON	S^*^_LN	S^*^_LC	S^*^_LS
Pure tree, DH	59	49.4	7.8	109.3	105.3	92.3
Tree, DH	71	56.9	8.6	236.0	131.5	127.0
Shrub, DH	39	33.4	5.0	62.4	83.7	65.4
Herb, DH	32	32.5	5.6	50.1	65.2	52.4
Pure tree, DK	80	58.2	8.3	116.6	166.8	127.3
Tree, DK	88	65.7	8.7	104.3	146.3	113.7
Shrub, DK	94	105.7	20.8	121.8	136.1	115.9
Herb, DK	47	55.7	10.6	45.8	65.8	51.5

*S* and S*: the observed and expected total number of species, respectively.

### Comparisons of LC-predicted SAD curves

**Fig 2:**
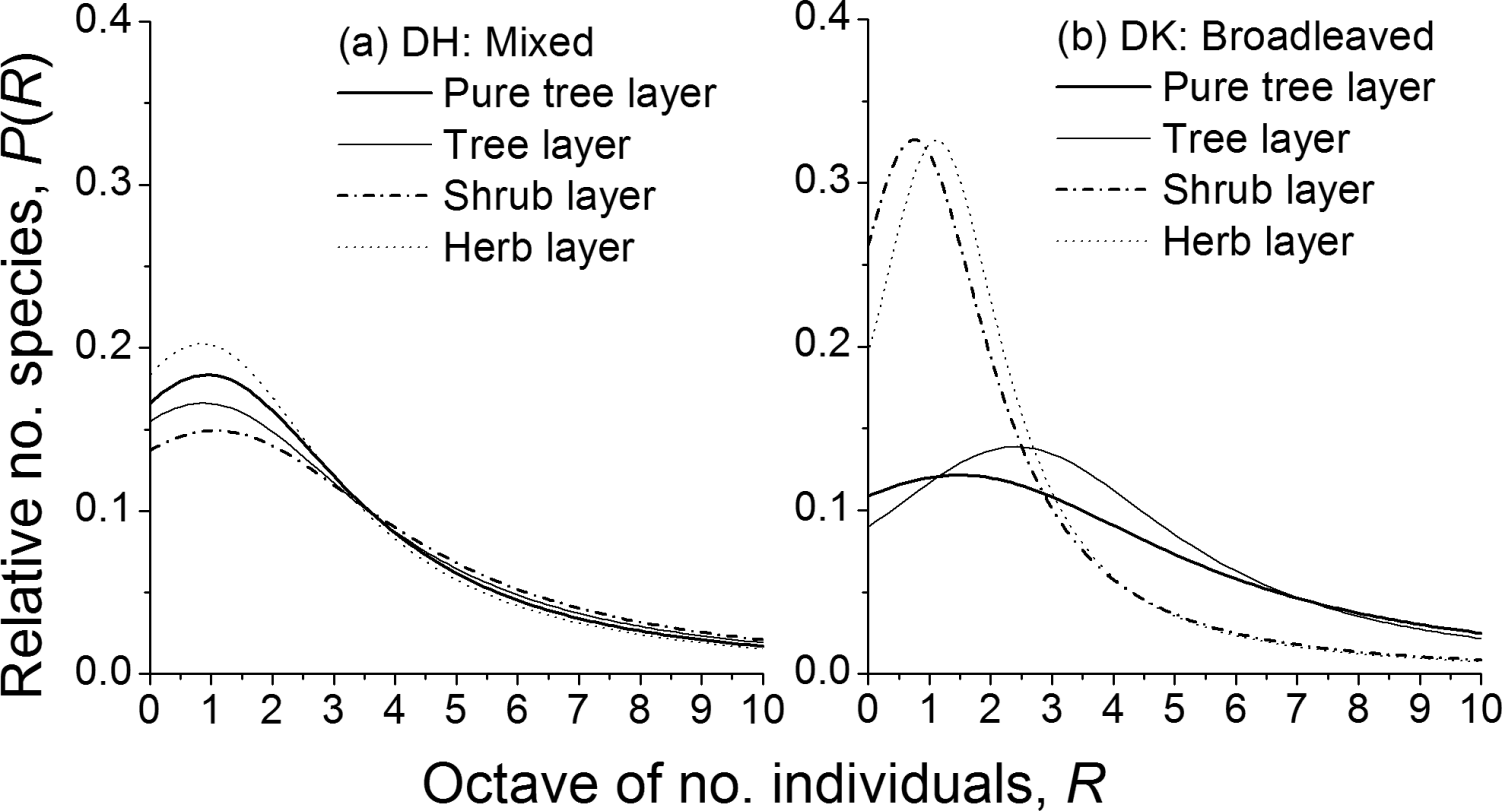
SADs predicted by left-truncated logCauchy (LC) models on octave (log_2_) scale in four layers of two communities in Dinghushan Biosphere Reserve, Guangdong of China. (Area below each distribution curve is set as unity).

As mentioned above, the LC model was the best in the set of seven models to agree with all the SAD data from four layers of two communities. So we plotted the probability distribution curves of LC-predicted SADs that were left-truncated (Fig 2). The curves comparatively showed the proportional number of species on the abundance octave for the different layers of different communities.

We first made the comparisons of different layers in the same community (Fig 2). The height of LC-predicted SAD curves of different layers showed different patterns. The order of curve height was herb layer > pure tree layer > tree layer > shrub layer for the mixed forest, while the order was herb layer > shrub layer > tree layer > pure tree layer for the broad-leaved forest. Except that the curve for the herb layer remained the highest, the order of curve height for the other three layers was just reversed from the mixed to the broad-leaved. This suggested that the commonness and rarity distribution of species changed with community succession: generally, the commonness moved up whereas the rarity moved down.

The curve height difference of the lower and upper layers between two forest communities also showed an interesting pattern (Fig 2). The curves of four layers were close to one another (i.e., the curves of lower layers were close to the ones of upper layers) in the mixed forest; while the curves of lower layers were much higher than the ones of upper layers in the broad-leaved forest. As a consequence, the height difference of SAD curves between the upper and the lower layers increased with the succession of the mixed to the broad-leaved forest. That is, the difference of species commonness and rarity distribution between the upper and the lower layers increased with succession.

The comparisons of fitted SADs were also made in the same layer between two communities (Fig 2). The height difference of probability curves for the lower layers was distinctly larger than the one for the upper layers between two communities. This suggested that the SADs of lower layers changed more than the ones of the upper layers with the succession. Besides, the order of curve height for the upper and lower layers between two communities was just reverse. The curve of the mixed forest was somewhat higher than the one of the broad-leaved for the upper layers, while the former was much lower than the latter for the lower layers. In other words, in the upper layers, the broad-leaved forest had more proportional common species (the right of curves) and less proportional rare species (the left of curves) than the mixed forest; while in the lower layers, the pattern was opposite. Therefore, with the succession of community, species’ commonness moved to the upper layers, but their rarity moved to the lower layers.

## Discussion

### Selection and mechanism of SAD models

We integrated the GS, BS, ON and LSer into the “sequential distribution set” composed of the LC, LS and LN [29], and produced a lager set of seven models by using model selection method [28]. The new set covers the empirical (statistical) and theoretical (niche) models and has very different function forms, so it has stronger modeling capability. Nevertheless, such a set of SAD models can be extended. As to the fitness order of models, the study was very similar to the previous studies [29,32]. This further proved that the LC model was the best in the set of either three or seven models. Yin et al. (2013) also fitted a set of 12 statistical distribution models to the datasets from four body size indicators (canopy diameter, breast-height diameter, ground diameter and tree height) and their six derived area and volume indicators of *Castanopsis hystrix* in Guangzhou, South China, and stated that the LC distribution was one of the best models which were commonly accepted [43]. So we conclude that the logCauchy model has the widest applicability in population and community ecology.

Ecologists proposed some mechanisms to interpret LN-typed SADs. The central limit theorem of statistics predicts that the LN distribution should produce when many variables interact multiplicatively [16,44–45]. Sugihara (1980) stated that the LN results from the species’ subdividing niche space within a community [3,13,46]. Engen & Lande (1996) derived the SAD of LN type from population dynamics models [47]. Hubbell’s (2001) neutral theory suggested that the SAD of LN-like shape is a particular case of his zero-sum multinomial distribution [48]. No matter what its origin is, the LN is important because it can predict the distribution of individual abundance among species (i.e., SAD, and thus S*), and has provided other valuable insights into the community organization such as species-area relationship and community succession [10–12,49–50]. The LC distribution should be also suitable for the scenarios of the LN because it has some characteristics similar to the latter [22,32].

### Pattern and dynamics of SAD

The SADs followed the GS model only for the shrub layer and herb layers (i.e., the lower layers) of the mixed forest in early successional stage, while they followed the LN (as well as the LC) for each layer of two forests in different successional stages. That is, whether for seven models to follow the observed was not directly related to the successional stage of community. This was somewhat different form Whittaker’s (1965) study that indicated that the SAD changed from the GS to the LN with the succession in an old field [15], but was similar to the results on the pattern and dynamics of SAD during the natural restoration of degraded hilly grassland in southern China [32].

For above reasons, we cannot qualitatively identify the community successional stage according to the SAD model type, and vice versa. However, we can quantitatively examine the relationship between the SAD pattern and community succession by using a universal model (here the LC). The LC-predicted SAD curves indicated that with the succession of forest community, the commonness of species moved up while the rarity of species moved down, and the difference of the commonness-rarity distribution of species increased between the upper and the lower layers. These results are confirmed with the study by Yin et al. (2009) [32]. With the development of community, the number of tree species increase and their spreading layers become higher and more, so the common species in the upper layers increase. Meanwhile, the understory plants (especially the herbs and the seedlings of trees and shrubs intolerant of shade) decrease with increasing canopy density, so the species rarity increases in the lower layers of community.

### Implications for studying SAD

He and Legendre (2002) stated that the area, abundance, richness, and spatial distribution of species have been central components of community ecology [51]. The quantitative studies on the structure and dynamics of a natural forest community can help solve some practical problems such as species selection and configuration in the reconstruction of forest vegetation [22,52]. This is because that the SAD can reveal the commonness-rarity of species, the proportional individual number of every species, and the distribution dynamics of different layers in natural communities. Our modeling results showed that the logCauchy model had the widest applicability and the best fitness to all the datasets among the seven models, and could best interpret the SAD patterns and dynamics of communities and their different layers in the succession of forests. These results will quantitatively provide feasible guidance for the reconstruction or improvement of public forests and the restoration of degraded vegetation. This study is also expected to enrich the theories in community ecology, macroecology, biogeography and restoration ecology.

## Conclusion

The SADs all showed left-truncated peak shape on log scale for the four layers of two forest communities in Dinghushan Biosphere Reserve, Guangdong of China. The model selection indicated that the LC model was the best among the set of seven models. We also found whether for the SAD models to follow the data was not directly related to the successional stage of forest community. Although we cannot qualitatively identify the successional stage of a community according to the type of SAD models and vice versa, we can quantitatively examine the changes of the SAD of community with succession through a commonly accepted model, the LC. The LC-predicted SAD curve showed that the commonness of species moved up while the rarity of species moved down with the succession of forest community. This is because that the broad-leaved forest had more proportional common species and less proportional rare species than the mixed forest in the upper layers, while the case was reverse in the lower layers. Moreover, the difference of the SADs between the upper and the lower layers increased with succession.

## Supporting information

S1 File The dataset necessary to replicate our study findings. (XLS)
